# Method comparison and estimation of causal effects of insomnia on health outcomes in a survey sampled population

**DOI:** 10.1038/s41598-023-36927-2

**Published:** 2023-06-17

**Authors:** Anja Shahu, Joon Chung, Wassim Tarraf, Alberto R. Ramos, Hector M. González, Susan Redline, Jianwen Cai, Tamar Sofer

**Affiliations:** 1 Department of Biostatistics, Harvard T.H. Chan of Public Health, Boston, MA USA; 2 Division of Sleep and Circadian Disorders, Department of Medicine, Brigham and Women’s Hospital, 221 Longwood Avenue, Boston, MA 02115 USA; 3 Institute of Gerontology, Wayne State University, Detroit, MI USA; 4 Department of Neurology, University of Miami Miller School of Medicine, Miami, FL USA; 5 Department of Neurosciences and Shiley-Marcos Alzheimer’s Disease Center, University of California, San Diego, La Jolla, CA USA; 6 Department of Biostatistics, University of North Carolina at Chapel Hill, Chapel Hill, NC USA; 7 CardioVascular Institute (CVI), Beth Israel Deaconness Medical Center, Boston, MA USA

**Keywords:** Epidemiology, Statistics, Risk factors

## Abstract

Applying causal inference methods, such as weighting and matching methods, to a survey sampled population requires properly incorporating the survey weights and design to obtain effect estimates that are representative of the target population and correct standard errors (SEs). With a simulation study, we compared various approaches for incorporating the survey weights and design into weighting and matching-based causal inference methods. When the models were correctly specified, most approaches performed well. However, when a variable was treated as an unmeasured confounder and the survey weights were constructed to depend on this variable, only the matching methods that used the survey weights in causal estimation and as a covariate in matching continued to perform well. If unmeasured confounders are potentially associated with the survey sample design, we recommend that investigators include the survey weights as a covariate in matching, in addition to incorporating them in causal effect estimation. Finally, we applied the various approaches to the Hispanic Community Health Study/Study of Latinos (HCHS/SOL) and found that insomnia has a causal association with both mild cognitive impairment (MCI) and incident hypertension 6–7 years later in the US Hispanic/Latino population.

## Introduction

Modifiable lifestyle behaviors, such as sleep, are essential to health, and are therefore targets for intervention to mitigate or prevent adverse health outcomes. While randomized controlled trials (RCTs) are the gold standard for causal inference, they can also be impractical and expensive and lack generalizability when using specific inclusion and exclusion criteria^1,2^. RCTs may also be unethical if they withhold treatment for some individuals when one is available^3^. Thus, researchers have called for greater use of causal inference methods in observational sleep studies to assess the potential impact of treatment effects^4^.

Using multiple causal inference methods can establish more robust causal associations than application of a single approach^5,6^. With the growing availability of complex health surveys conducted on racial and ethnic minorities, who have been historically underrepresented in research despite having higher disease burdens, investigators have more opportunities to make inferences on these populations and ensure that research is more representative of the world’s diversity^7,8^. However, complex health surveys––which use multi-stage probability sampling and include survey weights that contain information on the sampling design and adjustments for issues, such as non-response––present unique challenges. Survey weights and design must be incorporated into statistical models to obtain estimates representative of the target population and to provide correct standard errors (SEs)^9^. However, since causal inference methods were developed under the assumption of a simple random sample (SRS), incorporating the survey weights and design in a way that limits confounding while maintaining representativeness is not straightforward.

Motivated by the Hispanic Community Health Study/Study of Latinos (HCHS/SOL)––the largest longitudinal cohort study with multiple sleep measures at baseline and the only study with comprehensive sleep measures in a large, diverse sample of US Hispanics/Latinos, we aimed to investigate how to apply matching and weighting-based causal inference methods to complex health survey data. Both weighting and matching methods estimate the causal effect by balancing the distribution of covariates between the exposed and unexposed groups, relying on the three assumptions of exchangeability, positivity and Stable Unit Treatment Value Assumption (SUTVA)^10^. We conducted a simulation study to compare various approaches for incorporating the survey weights and design into weighting and matching methods^11–15^. We use the simulation results to inform our use of the HCHS/SOL for estimating the effect of insomnia on prevalent mild cognitive impairment (MCI) and incident hypertension in the US Hispanic/Latino population.

## Potential outcomes framework and causal estimands

Relying on a potential outcomes framework, suppose that a study has $n$ individuals sampled from a population of size $N$. An individual $i$ has two potential outcomes ${Y}_{i}\left(a\right)$, for exposure $a=0$ (unexposed) and $a=1$ (exposed)^16^. Let ${Z}_{i}$ be the indicator for observed exposure, with ${Z}_{i}=0$ if unexposed and ${Z}_{i}=1$ if exposed^16^. The individual’s observed outcome is then ${Y}_{i}\left({Z}_{i}\right)={Z}_{i}\times {Y}_{i}\left(1\right)+\left(1-{Z}_{i}\right)\times {Y}_{i}\left(0\right)$^16^.

At the population level, the average potential outcomes are represented by $E\left[Y\left(1\right)\right]$ and $E\left[Y\left(0\right)\right]$ when all individuals in the population are exposed and unexposed, respectively^17^. For binary outcomes, these values are represented by probabilities: $Pr\left[Y\left(1\right)=1\right]$ and $Pr\left[Y\left(0\right)=1\right]$, respectively^17^. Some causal effects of interest can include the rate difference $Pr\left[Y\left(1\right)=1\right]-Pr\left[Y\left(0\right)=1\right]$, the risk ratio $\frac{Pr\left[Y\left(1\right)=1\right]}{Pr\left[Y\left(0\right)=1\right]}$ and the odds ratio $\frac{\left(Pr\left[Y\left(1\right)=1\right]/Pr\left[Y\left(1\right)=0\right]\right)}{\left(Pr\left[Y\left(0\right)=1\right]/Pr\left[Y\left(0\right)=0\right]\right)}$^17^.

Common causal estimands (i.e., defined quantities that one can estimate from data) of interest include the average treatment effect (ATE), average treatment effect for the treated (ATT), conditional ATE (CATE) and conditional ATT (CATT)^16^. The marginal estimands, ATE and ATT, define exposure effect on the entire population and on those individuals who are observed as exposed, respectively^16^, obtained from analysis that is not adjusted for any covariates. The conditional estimands, CATE and CATT, align with the ATE and ATT definitions, but are additionally conditional on the sampling distribution of the covariates, ${X}_{i}$^16^, i.e. are obtained from analysis that adjusts for covariates. For a continuous outcome, we define ATE as $E\left[Y\left(1\right)-Y\left(0\right)\right]$, ATT as $E\left[Y\left(1\right)-Y\left(0\right)|Z=1\right]$, CATE as $E\left[Y\left(1\right)-Y\left(0\right)|X\right]$ and CATT as $E\left[Y\left(1\right)-Y\left(0\right)|Z=1, X\right]$^16^. Like the population causal effect, these definitions can be modified to apply to a binary outcome. In observational data that use exposure, rather than treatment, data, we use the term “exposed”, while in clinical trials and observational studied in which individuals are treated with a specific intervention, the term “treatment” is used. Henceforth we use “ATT” and “CATT” rather than “average exposure effect on the exposed” and “conditional average exposed effect on the exposed” for consistency with the causal inference literature.

The ATE and the ATT may coincide in a randomized controlled trial (RCT) due to randomization, but will not generally coincide in an observational study because the exposed and unexposed groups will not be comparable, i.e. they do not have the same characteristics and covariate distributions^18^. In an RCT, in the case of a continuous outcome, the ATE and CATE and the ATT and CATT will both coincide, i.e., the difference in continuous outcome means across treatment groups is “collapsible”. However, when the outcome is binary, these estimands may not coincide due to non-collapsibility^10^. Table 1 provides an overview of the causal inference methods that we compare and are described below, including information on the target estimand of each approach (ATE or ATT; and CATE or CATT if covariate adjusted).

**Table 1:** Comparison of weighting and matching-based causal inference methods.

	PSM	CEM	Weighting
Description	Match based on the propensity score to obtain a matched sample with balanced covariates	Bin based on coarsened variables to obtain a matched, weighted sample with balanced covariates	Use weights based on the propensity score to obtain a weighted sample with balanced covariates
ATT or ATE?	ATT^10^	ATT^19^	ATE for IPTW, ATT for weighting by the odds^10,18^
Package in R	MatchIt	MatchIt, cem	N/A

*ATE* average treatment effect, *ATT* average treatment effect for the treated, *CEM* coarsened exact matching, *IPTW* inverse probability of treatment weighting, *PSM* propensity score matching.

## Implementation of causal inference methods in a survey study

We study the application of two categories of causal inference approaches: matching and weighting methods. Briefly, matching methods typically identify sets (or minimally, pairs) of exposed and unexposed individuals who have similar characteristics and use these individuals in the regression analysis. Weighting methods perform weighted regression analysis, where each observation is weighted according to its probability of being exposed. Notably, this is an analogue of survey regression which weights each observation according to its sampling probability into the study (survey weight). A challenge of applying both matching and weighting-based causal inference methods to a survey-sampled population is in using the survey weights, which we call “original survey weights” (OSW), to obtain causal effect estimates that are representative of the target population.

Both matching and weighting methods may rely on both the OSW and on propensity score-based weights^10^. The propensity score for individual $i$ is defined as the probability of exposure, conditional on measured covariates: ${e}_{i}=P\left({Z}_{i}=1|{X}_{i1},\dots ,{X}_{ip}\right)$^10^. A popular method to calculate propensity scores is to use a logistic model given by ${\text{logit}}\left({e}_{i}\right)={\beta }_{0}+{\beta }_{1}{X}_{i1}+\dots +{\beta }_{p}{X}_{ip}$ where $p$ is the number of measured covariates^10^. For both the weighting and matching methods, we consider estimating the propensity scores in two ways: (1) OSW-weighted logistic regression, and (2) logistic regression with OSW as a covariate. In the weighting and matching methods sections below, we describe propensity score-based weights and additional method-specific weights.

### Matching methods

**Figure 1:**
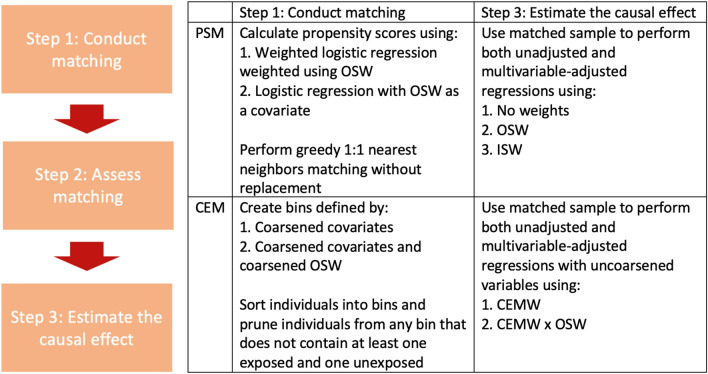
Steps in estimation of causal effects using the two compared matching methods: PSM and CEM. Left: the three steps in the estimation process. Right: comparison of the first and third steps between the two methods. Step 2 (assessing matching) compares means of covariates between the compared exposure groups using the weighting approaches described in step 3. *CEM* coarsened exact matching, *CEMW* coarsened exact matching weights, *ISW* inherited survey weights, *OSW* original survey weights, *PSM* propensity score matching.

Matching methods are generally implemented in three steps: (1) matching exposed and unexposed; (2) assessing covariate balance between the exposure groups and (3) estimating causal effect^10^. We studied both propensity score and coarsened exact matching (PSM and CEM) implemented using the “MatchIt” package in R. Generally, PSM matches individuals by ensuring that their propensity scores are similar; CEM first “coarsens” variables used for matching, with coarsening being the process of creating bins of values of continuous variables, followed by matching, i.e. ensuring that the coarsened variables are the same in matched individuals. We considered a few approaches, outlined in Fig. 1, to incorporating the survey weights and design in steps 1 and 3.

#### Matching exposed and unexposed

In PSM, we calculated the distance between individuals, defined as ${D}_{ij}={\widehat{e}}_{i}-{\widehat{e}}_{j}$^10^. We then used greedy 1:1 nearest neighbor matching without replacement. This algorithm matches every unexposed individual $i$ to the exposed individual with the smallest distance from individual $i$ and discards any unmatched unexposed individuals^10^. In CEM, we sorted individuals into bins based on coarsened variables^19^. We considered matching based on coarsened covariates only and based also on coarsened OSW. We coarsened the continuous covariates manually, choosing meaningful cut points when available or otherwise choosing quantiles as our cut points. We then pruned individuals from any bin that did not contain at least one exposed and one unexposed individual^19^. Specifically, the CEMW ${w}_{i}$ for individual $i$ is given by: ${w}_{i}={Z}_{i}+\left(1-{Z}_{i}\right)\left[\frac{{n}_{\mathrm{unexposed}}}{{n}_{\mathrm{exposed}}}\times \frac{{n}_{{b}_{i},\mathrm{exposed}}}{{n}_{{b}_{i},\mathrm{unexposed}}}\right]$, where ${b}_{i}$ is the bin that individual $i$ has been sorted into and ${n}_{\mathrm{unexposed}}$ and ${n}_{\mathrm{exposed}}$ are the numbers of unexposed and exposed individuals in the matched sample, respectively^20^. Thus, for matched individuals, the algorithm yielded CEMW that “equalize” the two groups of matched individuals by up- and down-weighting the number of exposed and unexposed individuals within each bin, and weight individuals in both groups so that both groups have similar characteristics to the exposed group^19,20^.

#### Estimating causal effects

For both PSM and CEM, we used the matched samples to fit Poisson regressions with a “log” link to estimate incident rate ratios (for incident outcomes) and logistic regressions to estimate odds ratios (for prevalent outcomes). We used both unadjusted and multivariable-adjusted regressions to estimate the marginal and conditional causal effects, respectively, incorporating the sampling design using the “survey” package in R for any weighted analysis. For PSM, we fit: (1) unweighted regression; (2) weighted with OSW and (3) weighted with inherited survey weights (ISW), in which unexposed individuals “inherit” the survey weight of the exposed individual that they are matched with. For CEM, we fit weighted regressions with: (1) CEMW and (2) CEMW $\times$ OSW.

### Weighting methods

We studied two types of propensity score-based weighting methods: (1) inverse probability of treatment weighting (IPTW), weighting both the exposed and unexposed individuals using their estimated exposure probabilities with ${w}_{i}=\frac{{Z}_{i}}{{\widehat{e}}_{i}}+\frac{1-{Z}_{i}}{1-{\widehat{e}}_{i}}$, and (2) weighting by the odds using ${w}_{i}={Z}_{i}+\left(1-{Z}_{i}\right)\frac{{\widehat{e}}_{i}}{1-{\widehat{e}}_{i}}$, where the unexposed are weighted by their odds of being exposed.

When estimating the causal effect, we fit Poisson regressions with a “log” link to estimate incident rate ratios (for incident outcomes) and logistic regressions to estimate odds ratios (for prevalent outcomes) on the full sample. These were weighted using: (1) propensity score weights (PSW) and (2) PSW $\times$ OSW, where PSW were either the IPTW or odds-weights above. We used both unadjusted and multivariable-adjusted weighted regressions, incorporating the sampling design using the “survey” package in R, to estimate the marginal and conditional causal effects, respectively.

### Assessment of matching and weighting

Metrics, such as the absolute standardized mean difference (SMD), can be compared before and after implementing weighting or matching methods to assess improvement in balance of covariates across the exposure groups^10,18^. We define the absolute SMD of a covariate as $\frac{\left|{\overline{x}}_{\mathrm{exposed}}-{\overline{x}}_{\mathrm{unexposed}}\right|}{{s}_{\mathrm{exposed}}}$, where ${\overline{x}}_{\mathrm{exposed}}$ and ${\overline{x}}_{\mathrm{unexposed}}$ are the means of covariate $x$ in the exposed and unexposed groups, and ${s}_{\mathrm{exposed}}$ is the standard deviation of $x$ in the full exposed group. In other words, the standard deviation ${s}_{\mathrm{exposed}}$ is computed using the full exposed group—before potentially sampling individuals for matching purposes—while accounting for survey design using weighting with OSW^10^. We similarly use OSW for weighting when estimating ${\overline{x}}_{\mathrm{exposed}}$ and ${\overline{x}}_{\mathrm{unexposed}}$. For categorical (including ordinal) variables, the absolute SMD for each level of the covariate is calculated, where now the mean of the covariate (at a given level) is the proportion of individuals with that level of the covariate, rather than treating the covariate as continuous^10,21^.

## Simulation study

### Sampling design

**Figure 2:**
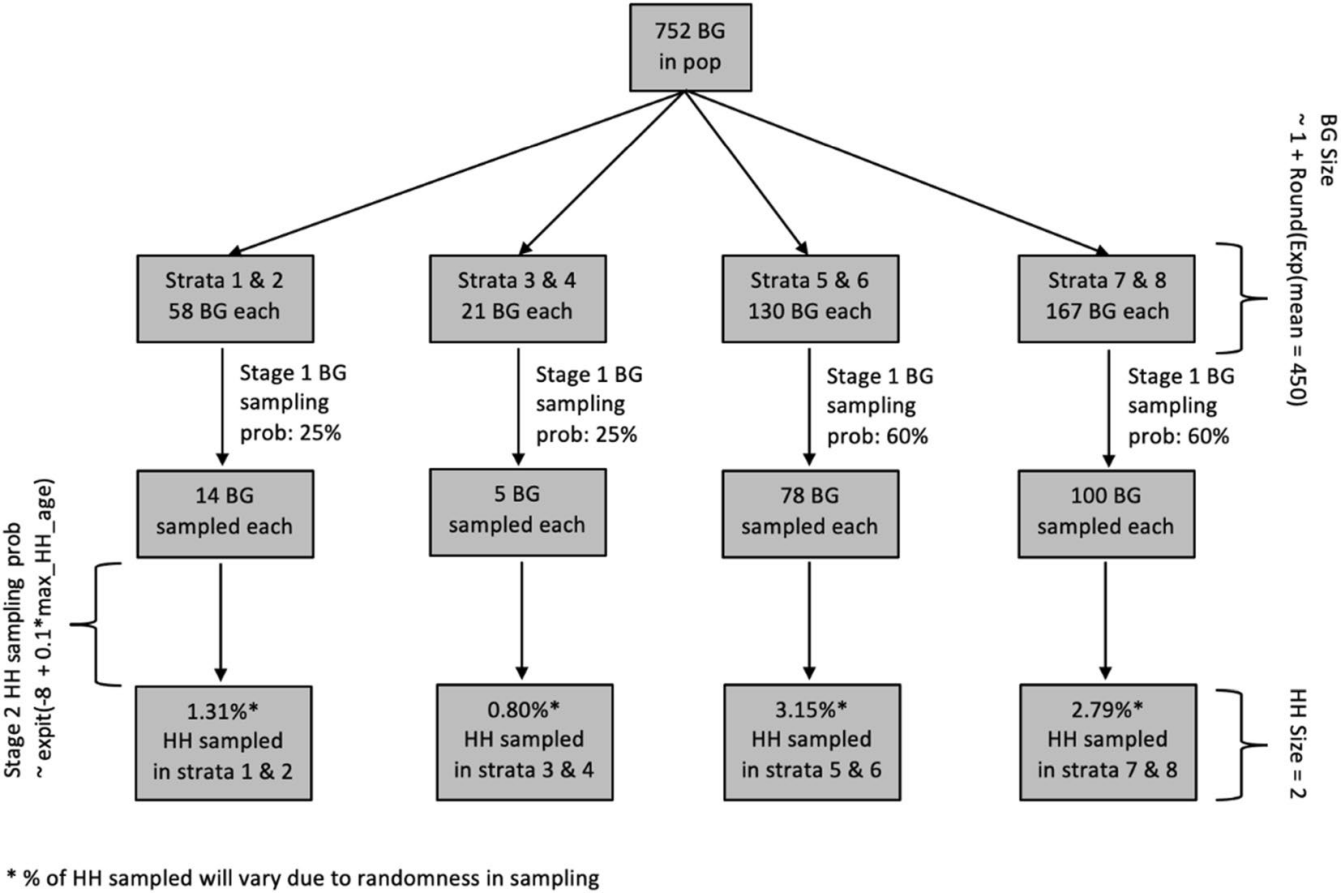
Flowchart illustrating sampling design from one sampled dataset for scenario 1, where survey weights are constructed to depend on the confounder, age. *BG* block group, *HH* household, *pop* population, *prob* probability.

We simulated complex health survey data with a nested structure, where the population was segmented into block groups (BGs), with equal-sized households (HHs) nested within the BGs. We used a stratified two-stage probability sampling design to draw 1000 independent samples from this population. This design mimicked the sampling design of the Bronx site in the HCHS/SOL^22^. Figure 2 provides an overview of the sampling design. The population contained 752 BGs split unevenly across 8 strata. We assigned the BGs strata-specific sampling probabilities. The BG sampling probability was 25% for BGs in strata 1–4 and 60% for BGs in strata 5–8. We sampled entire BGs without replacement from the population based on these strata-specific BG sampling probabilities.

In the primary scenario 1 (Fig. 2), we generated the number of HHs to vary for each BG using an exponential distribution with mean of 450. Within each HH, we generated 2 individuals and their ages, and set the HH sampling probabilities to depend on the maximum age of the HH. First, we sampled a mean age for the HH as $N\left({\mathrm{40,15}}^{2}\right)$, truncated to a range of 23 to 69. Second, we sampled the age of the first individual and second individual from a uniform, discrete distribution that ranged within 10 years of the mean age. For each HH, the HH sampling probability was calculated as ${\text{expit}}\left(-8+0.1\times \text{max\_HH\_age}\right)$, where ${\text{expit}}\left(x\right)=\frac{\mathrm{exp}\left(x\right)}{1+\mathrm{exp}\left(x\right)}$. From the BGs that were selected in stage 1, we sampled equal-sized HHs without replacement based on these HH sampling probabilities. In a secondary scenario 2, we did not use age in the sampling design (Supplementary Fig. 1).

We calculated survey weights for each sample in three steps. We let $i$ designate the BG, $j$ designate the HH and $k$ designate the individual. First, we calculated the individual sampling probability as ${{p}_{ijk}=p}_{i}{p}_{ij}$, where ${p}_{i}$ is the BG sampling probability and ${p}_{ij}$ is the HH sampling probability. Second, we calculated the base weights as ${w}_{ijk}=\frac{1}{{p}_{ijk}}$. Third, we calculated the final weights to use in our analyses as ${W}_{ijk}=\frac{{w}_{ijk}}{\frac{1}{n}\sum_{i,j,k}{w}_{ijk}}$.

### Generating variables and association models

According to the description below, we generated the following variables: BMI and years between visits as predictors; insomnia as the exposure of interest; hypertension status in visits 1 and 2 and MCI in visit 2 as outcomes. In brief, we generated the outcomes for a visit using a potential outcomes framework, i.e. by simulating the outcomes under two (observed and unobserved) exposure values, to allow estimation of both the true marginal and conditional population causal effects.

In detail, in addition to age, we generated two other predictors, baseline BMI and years between visits. BMI and years between visits were generated independently for all individuals using $N\left({\mathrm{29,9}}^{2}\right)$, truncated to the range of 15 to 63, and using $N\left({\mathrm{6,0.5}}^{2}\right)$, truncated to the range of 3 to 9, respectively.

We generated the binary exposure, insomnia, independently for all individuals in two steps. First, we calculated the probability that an individual has insomnia using the following logistic model:

$${\text{logit}}\left(\mathit{Pr}\left({Z}_{i}=1\right)\right)={\alpha }_{0}+{\alpha }_{1}bm{i}_{i}+{\alpha }_{2}ag{e}_{i},$$

where ${\alpha }_{0}=\text{log(}0.109)$, ${\alpha }_{1}=\text{log(}1.025)$ and ${\alpha }_{2}=\text{log(}1.019)$, inferred from the HCHS/SOL data. Second, we used $\mathit{Pr}\left({Z}_{i}=1\right)$ to sample the observed insomnia status, ${Z}_{i}$, from a Bernoulli distribution.

For the binary outcomes, we generated prevalent MCI that was measured at visit 2 only and incident hypertension that was measured at both visit 1 and 2. Both outcomes were generated based on the HCHS/SOL data so that the prevalence of hypertension at each visit was relatively high ($\approx$ 40%), while the prevalence of MCI was low ($\approx$ 8%).

We generated the outcomes for a visit using a potential outcomes framework that consisted of three steps to allow estimation of both the true marginal and conditional population causal effects. For an individual, let ${Y}_{ijk1}$ designate the outcome at visit 1 and ${Y}_{ijk2}$ designate the outcome at visit 2. Let ${h}_{ij}$ be the HH clustering effect generated using $N\left(\mathrm{0,1}\right)$ and ${b}_{i}$ be the BG clustering effect generated using $N\left({\mathrm{0,0.5}}^{2}\right)$. First, for a visit, we calculated the potential probabilities of the outcome under $\mathrm{a}=1$ (insomnia) and $\mathrm{a}=0$ (no insomnia) using logistic regression models.

For prevalent MCI at visit 2, we used the following model:

$${\mathrm{logit}\left(\mathrm{Pr}\left[{Y}_{ijk2}\left(a\right)=1\right]\right)=\beta }_{0}+{\beta }_{1}a+{\beta }_{2}bm{i}_{ijk}+{\beta }_{3}ag{e}_{ijk}+{h}_{ij}+{b}_{i},$$

where ${\beta }_{0}=\mathrm{log}\left(0.003\right)$, ${\beta }_{1}=\mathrm{log}\left(1.560\right)$, ${\beta }_{2}=\mathrm{log}\left(1.018\right)$ and ${\beta }_{3}=\mathrm{log}\left(1.056\right)$, based on the HCHS/SOL data.

For hypertension status at visit 1 and visit 2, we used the following models:

$${\mathrm{logit}\left(\mathrm{Pr}\left[{Y}_{ijk1}\left(a\right)=1\right]\right)=\gamma }_{0}+{\gamma }_{1}a+{\gamma }_{2}bm{i}_{ijk}+{\gamma }_{3}ag{e}_{ijk}+{h}_{ij}+{b}_{i},$$

$${\mathrm{logit}\left(\mathrm{Pr}\left[{Y}_{ijk2}\left(a\right)=1\right]\right)=\phi }_{0}+{\phi }_{1}a+{\phi }_{2}bm{i}_{ijk}+{\phi }_{3}ag{e}_{ijk}+{\phi }_{4}year{s}_{ijk}+{h}_{ij}+{b}_{i},$$

where ${\gamma }_{0}=\mathrm{log}\left(0.002\right)$, ${\gamma }_{1}=\mathrm{log}\left(1.065\right)$, ${\gamma }_{2}=\mathrm{log}\left(1.088\right)$, ${\gamma }_{3}=\mathrm{log}\left(1.082\right)$, ${\phi }_{0}=\mathrm{log}\left(0.001\right)$, ${\phi }_{1}=\mathrm{log}\left(1.247\right)$, ${\phi }_{2}=\mathrm{log}\left(1.082\right)$, ${\phi }_{3}=\mathrm{log}\left(1.092\right)$ and ${\phi }_{4}=\mathrm{log}\left(1.098\right)$, based on the HCHS/SOL data.

Second, we used the respective probabilities to sample ${Y}_{ijk1}\left(\mathrm{a}\right)$ and ${Y}_{ijk2}\left(\mathrm{a}\right)$ from Bernoulli distributions under $a=1$ and $a=0$. Third, we identified the outcomes that were observed under ${Z}_{i}$.

In a sensitivity simulation analysis, we generated a new variable which we named education. We replaced age with education in the data generating models for insomnia, MCI, and hypertension. Education was generated for an individual in two steps, while ensuring that it is correlated with age. First, we drew from $Unif(min\left(age\right), max\left(age\right))$. Then, we drew from a Bernoulli distribution to decide if that value should be replaced with the individual’s age. The Bernoulli probability was chosen such that education would be correlated with age with correlation $\rho \in \{0.25, 0.5, 0.75\}$.

### Calculating true causal effects

We estimated the true marginal and conditional causal effects for the population of size $N$ in two steps. First, we created a new data frame with $2N$ observations, in which every individual has an observation for each potential outcome. Second, using the new data frame, we fit multiple regression models, each targeting a separate causal estimand. Specifically, we estimated the ATE and the CATE using the complete new data frame, as well as the ATT and CATT using only the observations where ${Z}_{i}=1$. For prevalent MCI, we fit marginal logistic regressions (regressing MCI on insomnia; estimating ATE and ATT) and conditional logistic regressions (regressing MCI on insomnia, BMI and age; estimating CATE and CATT). For incident hypertension, using a “log” link, we fit marginal Poisson regressions (regressing hypertension on insomnia with log of years between visits included as an offset; estimating ATE and ATT) and conditional Poisson regressions (regressing hypertension on insomnia, BMI and age with the log of years between visits included as an offset; estimating CATE and CATT) on the observations that did not have hypertension at baseline. For both outcomes, we used the exponentiated coefficient estimates on insomnia as the true causal effects.

### Performance measures

We used bias and 95% confidence interval (CI) coverage to compare the different approaches to using the survey weights and design on the simulated data. We calculated bias as $\frac{1}{1000}\sum_{i=1}^{1000}({\widehat{T\mathrm{E}}}_{\mathrm{i}}-TE)$ where $1000$ was the number of samples that were drawn from our simulated population, $TE$ was the true causal effect and ${\widehat{TE}}_{i}$ was the estimated causal effect for the $i$th sample. We calculated 95% CI coverage as the percentage of simulated samples with a 95% CI that contained the true causal effect: $100\times \frac{1}{1000}\sum_{i=1}^{1000}I\left(TE\in C{I}_{i}\right)$ where $C{I}_{i}$ was the 95% CI for the $i$th sample. An approach performs well when it has low bias and coverage near 95%.

### Sensitivity analyses

We performed three types of sensitivity analyses. One, for both scenarios 1 and 2, we treated age as an unmeasured confounder and re-ran the analyses to assess sensitivity to omission of confounding variables that are correlated with the survey weights. Two, we then further focused on the analysis methods that had good performance in this scenario 1 sensitivity analysis, and generated another confounding variable named (without loss of generality) education, and used it instead of age in the data generating models for insomnia and for the outcomes (MCI and hypertension). We generated this variable so that it is correlated with age with varying degrees of correlation ($\rho \in \{0.25, 0.5, 0.75\}$). In this setting, age was still a design variable. Thus, we assessed the degree to which correlation of an unmeasured confounder with a design variable may help recover the underlying causal effect size. Three, for scenario 1, we re-generated insomnia, MCI and hypertension multiple times by varying the model intercepts and re-ran the analyses to assess sensitivity to changes in the prevalence of the exposure and outcomes. The intercepts were chosen so that the prevalence of the exposure and outcome varied from 5 to 35 in increments of 10.

### Results

Tables 2 and 3 and Supplementary Tables 1 and 2 provide the simulation results of the various approaches to incorporating the survey weights and design into the matching and weighting methods, respectively. Under correct specification of the matching and weighting approaches, all approaches, excluding the PSM approaches using ISW, performed well for prevalent MCI and incident hypertension in both scenarios 1 and 2 (without age in the sampling design). When age was omitted from the matching and effect estimation models (i.e. under-specification), most approaches experienced increases in bias and poor coverage. In scenario 2, no approach performed well. However, in scenario 1, methods that used OSW as a covariate in matching or the propensity score calculation, in addition to incorporating OSW during causal effect estimation, continued to perform well.

**Table 2:** Simulation results for estimating effect of insomnia on prevalent MCI using various matching methods in the two compared scenarios.

					Scenario 1		Scenario 2	
Specification	Method	Matching	Adjustment	Weights	Bias	Cover	Bias	Cover
Correct	PSM	PS via weighted logistic regression using OSW	Unadjusted	No weights	− 0.004	0.969	0.011	0.951
				OSW	0.018	0.939	0.016	0.940
				ISW	− 0.266	0.420	0.015	0.944
			Adjusted	No weights	− 0.019	0.955	0.007	0.953
				OSW	0.014	0.936	0.014	0.932
				ISW	0.010	0.941	0.014	0.943
		PS via logistic regression with OSW as covariate	Unadjusted	No weights	− 0.007	0.969	0.009	0.951
				**OSW**	**0.018**	**0.946**	**0.015**	**0.943**
				ISW	− 0.267	0.407	0.013	0.927
			Adjusted	No weights	− 0.020	0.964	0.006	0.941
				**OSW**	**0.019**	**0.937**	**0.014**	**0.940**
				ISW	0.009	0.943	0.015	0.934
	CEM	Binning via coarsened covariates	Unadjusted	CEMW	0.045	0.906	0.048	0.909
				CEMW × OSW	0.033	0.948	0.052	0.910
			Adjusted	CEMW	− 0.021	0.954	0.005	0.954
				CEMW × OSW	0.009	0.944	0.011	0.943
		Binning via coarsened covariates and OSW	Unadjusted	CEMW	0.041	0.915	0.047	0.910
				**CEMW** × **OSW**	**0.030**	**0.947**	**0.052**	**0.910**
			Adjusted	CEMW	− 0.021	0.957	0.004	0.950
				**CEMW** × **OSW**	**0.009**	**0.945**	**0.011**	**0.950**
Under (no age)	PSM	PS via weighted logistic regression using OSW	Unadjusted	No weights	0.138	0.502	0.271	0.243
				OSW	0.279	0.563	0.275	0.410
				ISW	− 0.573	0.002	0.275	0.405
			Adjusted	No weights	0.114	0.648	0.249	0.342
				OSW	0.254	0.634	0.252	0.485
				ISW	− 0.598	0.002	0.252	0.491
		PS via logistic regression with OSW as covariate	Unadjusted	No weights	0.067	0.846	0.267	0.285
				**OSW**	**0.045**	**0.937**	**0.273**	**0.413**
				ISW	− 0.330	0.164	0.272	0.417
			Adjusted	No weights	0.046	0.906	0.245	0.347
				**OSW**	**0.020**	**0.948**	**0.250**	**0.500**
				ISW	− 0.420	0.038	0.250	0.482
	CEM	Binning via coarsened covariates	Unadjusted	CEMW	0.146	0.366	0.279	0.115
				CEMW × OSW	0.286	0.469	0.284	0.234
			Adjusted	CEMW	0.114	0.567	0.245	0.208
				CEMW ×	0.253	0.573	0.251	0.348
		Binning via coarsened covariates and OSW	Unadjusted	CEMW	0.054	0.885	0.279	0.116
				**CEMW** × **OSW**	**0.050**	**0.937**	**0.285**	**0.232**
			Adjusted	CEMW	0.022	0.943	0.245	0.211
				**CEMW** × **OSW**	**0.017**	**0.949**	**0.251**	**0.352**

Scenario 1: survey weights depend on age. Scenario 2: survey weights do not depend on age. Bias: difference between the causal effect estimate obtained from the full (unsampled) simulated target population dataset and the average estimated causal effect over the 1000 survey samples. Cover: coverage of the 95% CIs defined as the proportion of simulations in which the true effect is covered by the CIs.

*CEM* coarsened exact matching, *CEMW* coarsened exact matching weights, *CI* confidence interval; *Cover* coverage, *ISW* inherited survey weights, *MCI* mild cognitive impairment, *OSW* original survey weights, *PS* propensity score, *PSM* propensity score matching.

The four matching methods that we consider robust based on results from simulations with under specification are highlighted with bold text.

**Table 3:** Simulation results for estimating effect of insomnia on incident hypertension using various matching methods in the two compared scenarios.

					Scenario 1		Scenario 2	
Specification	Method	Matching	Adjustment	Weights	Bias	Cover	Bias	Cover
Correct	PSM	PS via weighted logistic regression using OSW	Unadjusted	No weights	− 0.036	0.942	− 0.004	0.982
				OSW	− 0.001	0.941	− 0.001	0.946
				ISW	− 0.087	0.776	− 0.002	0.953
			Adjusted	No weights	− 0.031	0.969	− 0.001	0.993
				OSW	0.002	0.934	0.002	0.949
				ISW	0.004	0.945	0.001	0.940
		PS via logistic regression with OSW as covariate	Unadjusted	No weights	− 0.037	0.948	− 0.005	0.986
				**OSW**	− **0.001**	**0.956**	− **0.002**	**0.958**
				ISW	− 0.079	0.784	− 0.004	0.946
			Adjusted	No weights	− 0.031	0.978	− 0.001	0.993
				**OSW**	**0.004**	**0.942**	**0.002**	**0.958**
				ISW	0.009	0.934	0.002	0.947
	CEM	Binning via coarsened covariates	Unadjusted	CEMW	− 0.020	0.901	0.009	0.961
				CEMW × OSW	0.002	0.954	0.012	0.947
			Adjusted	CEMW	− 0.034	0.732	− 0.005	0.953
				CEMW × OSW	− 0.002	0.939	− 0.002	0.961
		Binning via coarsened covariates and OSW	Unadjusted	CEMW	− 0.021	0.890	0.009	0.962
				**CEMW** × **OSW**	**0.002**	**0.946**	**0.012**	**0.953**
			Adjusted	CEMW	− 0.034	0.744	− 0.005	0.955
				**CEMW** × **OSW**	− **0.002**	**0.933**	− **0.002**	**0.962**
Under (no age)	PSM	PS via weighted logistic regression using OSW	Unadjusted	No weights	0.047	0.911	0.122	0.340
				OSW	0.126	0.707	0.126	0.330
				ISW	− 0.345	0.002	0.124	0.351
			Adjusted	No weights	0.050	0.899	0.127	0.313
				OSW	0.128	0.708	0.130	0.310
				ISW	− 0.360	0.000	0.129	0.318
		PS via logistic regression with OSW as covariate	Unadjusted	No weights	− 0.009	0.994	0.123	0.350
				**OSW**	− **0.001**	**0.952**	**0.127**	**0.355**
				ISW	− 0.052	0.915	0.122	0.372
			Adjusted	No weights	− 0.004	0.996	0.128	0.316
				**OSW**	− **0.008**	**0.952**	**0.131**	**0.310**
				ISW	− 0.154	0.473	0.128	0.311
	CEM	Binning via coarsened covariates	Unadjusted	CEMW	0.050	0.608	0.131	0.081
				CEMW × OSW	0.134	0.627	0.133	0.167
			Adjusted	CEMW	0.049	0.607	0.126	0.097
				CEMW × OSW	0.128	0.656	0.128	0.194
		Binning via coarsened covariates and OSW	Unadjusted	CEMW	− 0.013	0.926	0.130	0.087
				**CEMW** × **OSW**	**0.013**	**0.950**	**0.133**	**0.170**
			Adjusted	CEMW	− 0.017	0.919	0.126	0.094
				**CEMW** × **OSW**	**0.003**	**0.951**	**0.128**	**0.195**

Scenario 1: survey weights depend on age. Scenario 2: survey weights do not depend on age. Bias: difference between the causal effect estimate obtained from the full (unsampled) simulated target population dataset and the average estimated causal effect over the 1000 survey samples. Cover: coverage of the 95% CIs defined as the proportion of simulations in which the true effect is covered by the CIs.

*CEM* coarsened exact matching, *CEMW* coarsened exact matching weights, *CI* confidence interval, *Cover* coverage, *ISW* inherited survey weights, *MCI* mild cognitive impairment, *OSW* original survey weights, *PS* propensity score, *PSM* propensity score matching.

The four matching methods that we consider robust based on results from simulations with under specification are highlighted with bold text.

Highlighted in Tables 2 and 3 are four matching approaches identified as robust based on two subjective criteria: (1) coverage between 93 and 97% for scenarios 1 and 2 under correct specification; and (2) coverage between 93 and 97% for scenario 1 during under-specification. The robust PSM methods used propensity score computed via logistic regression with OSW as a covariate for matching, and next fitted regressions weighted using OSW. The robust CEM methods conducted matching using both coarsened covariates and coarsened OSW, following by regressions weighted using CEMW × OSW.

Table 4 provides results from the sensitivity analysis in which a confounder (education) was correlated with one of the survey design variables (age) and compares estimation results with and without including education in the analysis (correct specification and under-specification, respectively), by degree of the correlation between age and education. This sensitivity analysis focuses on the four robust matching methods identified in the first sensitivity analysis above. When education is not incorporated in the analysis, we see that the higher its correlation is with the design variable, the better the robust methods are able to recover the underlying causal effect.

**Table 4:** Simulation results from the second sensitivity analysis using the four robust matching methods to assess the degree to which correlation of an unmeasured confounder with a design variable may help recover the underlying causal effect size.

					Corr = 0.25		Corr = 0.50		Corr = 0.75	
Specification	Method	Matching	Adjustment	Weights	Bias	Cover	Bias	Cover	Bias	Cover
Outcome: prevalent MCI										
Correct	PSM	PS via logistic regression with OSW as covariate	Unadjusted	OSW	0.018	0.952	0.023	0.935	− 0.020	0.959
			Adjusted		0.035	0.945	0.036	0.930	− 0.004	0.953
	CEM	Binning via coarsened covariates and OSW	Unadjusted	CEMW × OSW	0.037	0.943	0.041	0.933	0.004	0.955
			Adjusted		0.035	0.939	0.034	0.932	− 0.013	0.946
Under (no education)	PSM	PS via logistic regression with OSW as covariate	Unadjusted	OSW	0.316	0.607	0.248	0.685	0.117	0.900
			Adjusted		0.314	0.609	0.241	0.705	0.102	0.913
	CEM	Binning via coarsened covariates and OSW	Unadjusted	CEMW × OSW	0.320	0.486	0.258	0.583	0.122	0.862
			Adjusted		0.309	0.518	0.245	0.613	0.101	0.897
Outcome: incident hypertension										
Correct	PSM	PS via logistic regression with OSW as covariate	Unadjusted	OSW	0.006	0.957	0.007	0.952	0.002	0.958
			Adjusted		0.011	0.945	0.008	0.958	0.003	0.940
	CEM	Binning via coarsened covariates and OSW	Unadjusted	CEMW × OSW	0.019	0.940	0.017	0.961	0.013	0.962
			Adjusted		0.011	0.934	0.007	0.950	− 0.001	0.940
Under (no education)	PSM	PS via logistic regression with OSW as covariate	Unadjusted	OSW	0.152	0.614	0.110	0.748	0.056	0.920
			Adjusted		0.155	0.593	0.107	0.757	0.050	0.926
	CEM	Binning via coarsened covariates and OSW	Unadjusted	CEMW × OSW	0.163	0.486	0.122	0.660	0.069	0.848
			Adjusted		0.161	0.490	0.116	0.671	0.061	0.871

The simulations were performed under Scenario 1. Corr: correlation between age and education. Bias: difference between the causal effect estimate obtained from the full (unsampled) simulated target population dataset and the average estimated causal effect over the 1000 survey samples. Cover: coverage of the 95% CIs defined as the proportion of simulations in which the true effect is covered by the CIs.

*CEM* coarsened exact matching, *CEMW* coarsened exact matching weights, *CI* confidence interval, *Cover* coverage, *ISW* inherited survey weights, *MCI* mild cognitive impairment, *OSW* original survey weights, *PS* propensity score, *PSM* propensity score matching.

**Figure 3:**
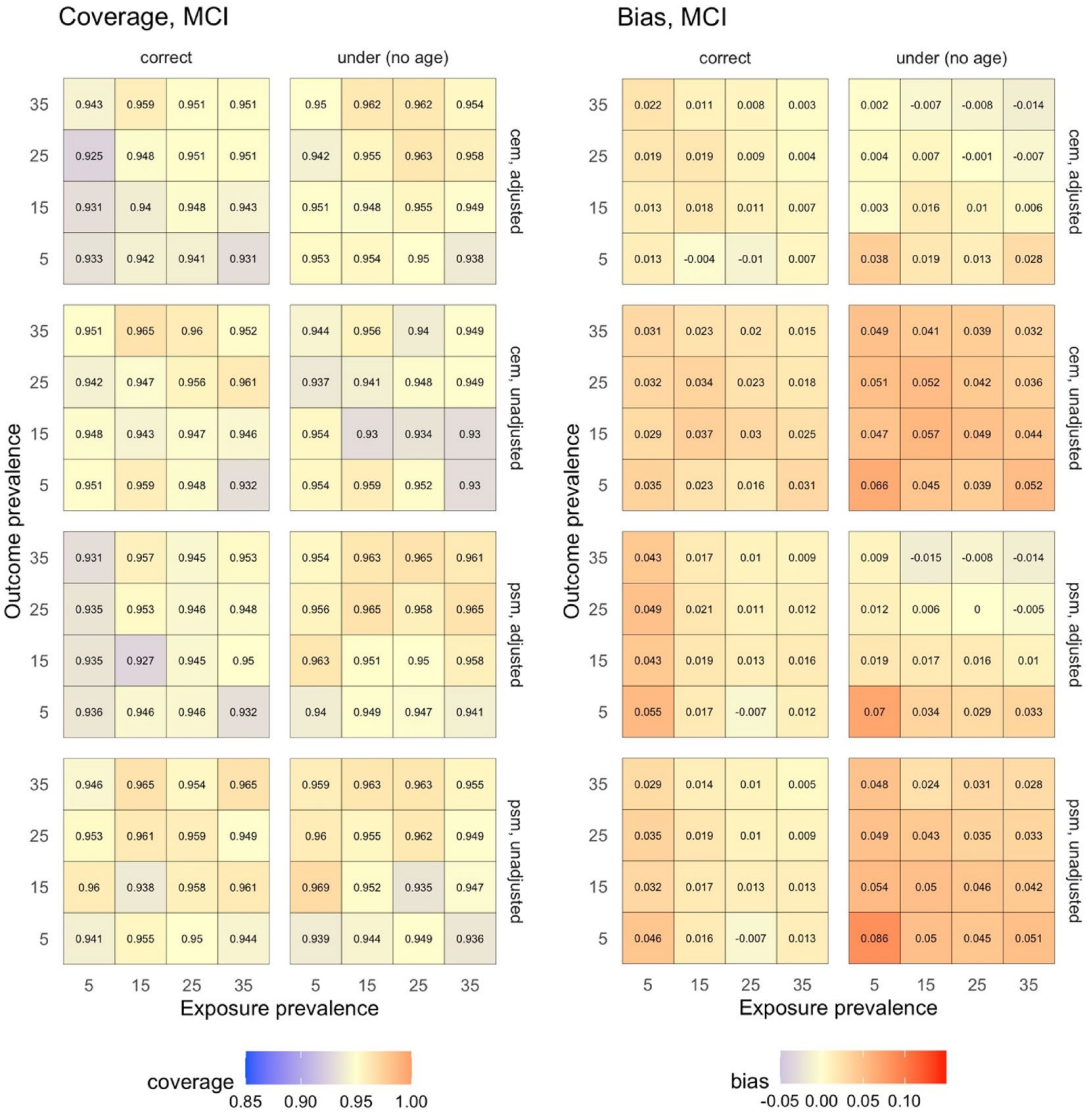
Simulation results for sensitivity analysis conducted on the robust matching methods to assess the effect of varying the prevalence of both the exposure and the outcome on coverage (left) and bias (right) during estimation of the effect of insomnia (exposure) on prevalent MCI (outcome). *CEM* coarsened exact matching, *MCI* mild cognitive impairment, *PSM* propensity score matching.

**Figure 4:**
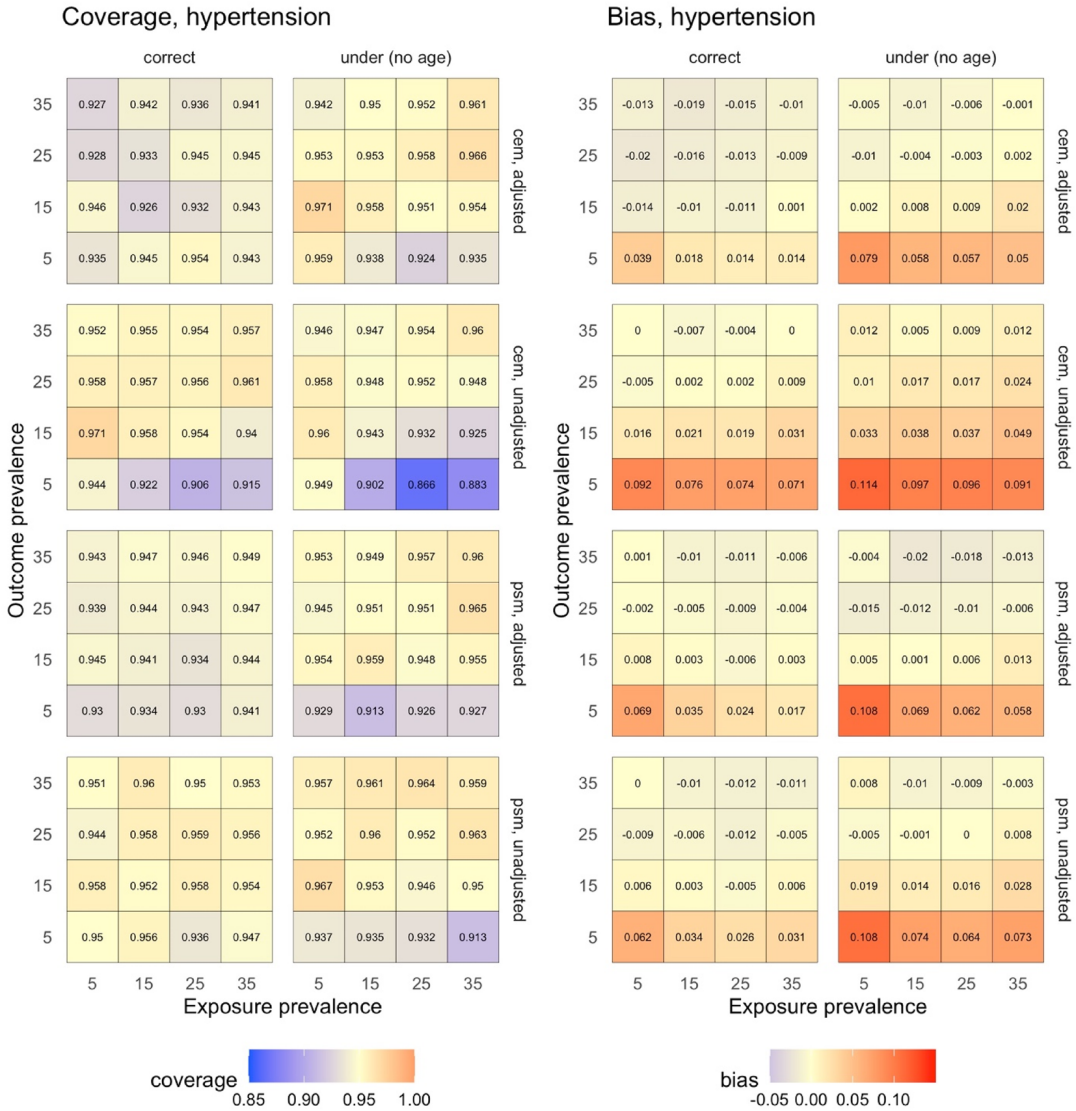
Simulation results for sensitivity analysis conducted on the robust matching methods to assess the effect of varying the prevalence of both the exposure and the outcome on coverage (left) and bias (right) during estimation of the effect of insomnia (exposure) on incident hypertension (outcome). *CEM* coarsened exact matching, *MCI* mild cognitive impairment, *PSM* propensity score matching.

Figures 3 and 4 provide the results of the sensitivity analysis to assess the effect of changing the exposure and outcome prevalences on the identified robust matching methods. Both bias and coverage appear robust to changes in the exposure and outcome prevalences as long as the prevalences are not rare (i.e. > 5%).

## Data analysis

### Hispanic community health study/study of latinos

The HCHS/SOL is a community based, multi-center, longitudinal cohort study of Hispanic/Latinos in the US^23^. A goal of the study was to investigate causal risk factors of diseases in Hispanic/Latino individuals^23^. In 2008, the study recruited over 16,415 men and women, aged 18–74, who self-identified as Hispanic/Latino, from four communities: Bronx, NY; Chicago, IL; Miami, FL and San Diego, CA^23^. HCHS/SOL is a complex health survey with a stratified three-stage probability sample^24^. Investigators used unequal sampling probabilities in each stage, selecting census BGs in stage 1, households in stage 2 and individuals in stage 3, and prioritized sampling of households more likely to have adults ages 45–74^24^.

The HCHS/SOL was approved by the institutional review boards (IRBs) at each field center, where all participants gave written informed consent in their preferred language (Spanish/English), and by the Non-Biomedical IRB at the University of North Carolina at Chapel Hill, to the HCHS/SOL Data Coordinating Center. All IRBs approving the study are: Non-Biomedical IRB at the University of North Carolina at Chapel Hill. Chapel Hill, NC; Einstein IRB at the Albert Einstein College of Medicine of Yeshiva University. Bronx, NY; IRB at Office for the Protection of Research Subjects (OPRS), University of Illinois at Chicago. Chicago, IL; Human Subject Research Office, University of Miami. Miami, FL; Institutional Review Board of San Diego State University, San Diego, CA. The study reported here was approved by the Mass General Brigham IRB under protocol #2022P001237. All methods were carried out in accordance with relevant guidelines and regulations.

### Exposure and predictors

Insomnia was defined using the Women Health Initiative Insomnia Rating Scale (WHIIRS) ≥ 9^25^. The other included predictors were: time between visits; Hispanic/Latino background (Dominican, Central American, Cuban, Mexican, Puerto Rican, South American, more than one/other heritage); alcohol (never, former, current); smoking (never, former, current); age; gender (female, male); marital status (married or living with partner, single, separated, divorced or widower); education (no high school diploma or GED, at most a high school diploma or GED, greater than high school diploma or GED); BMI; employment (retired and not currently employed or missing on employment, not retired or missing on retirement and not currently employed, employed part-time, < 35 h/week, employed full-time, > 35 h/week). Table 5 provides a summary of the predictors stratified by insomnia status.

**Table 5:** Demographics and BMI of HCHS/SOL stratified by insomnia status.

	Incident hypertension sample			Prevalent MCI sample		
	No insomnia (N = 4092)	Insomnia (N = 2005)	Total (N = 6097)	No insomnia (N = 3580)	Insomnia (N = 2506)	Total (N = 6086)
Hispanic/Latino background, %						
Dominican	7.5	10.8	8.5	7.7	11.6	9.2
Central American	7.8	7.0	7.6	7.8	6.7	7.4
Cuban	14.0	16.3	14.7	25.8	25.4	25.7
Mexican	50.7	38.9	47.2	37.0	27.6	33.3
Puerto Rican	9.7	18.4	12.3	11.9	21.2	15.6
South American	6.2	4.4	5.7	5.9	3.9	5.1
More than one/other	4.0	4.1	4.0	3.8	3.6	3.7
Alcohol, %						
Never	19.1	15.3	18.0	23.5	22.3	23.0
Former	29.0	33.4	30.3	29.5	34.0	31.3
Current	51.9	51.3	51.7	47.0	43.6	45.7
Smoking, %						
Never	68.6	59.6	65.9	56.0	54.1	55.3
Former	13.3	16.2	14.2	27.0	24.6	26.1
Current	18.1	24.2	20.0	17.0	21.2	18.6
Age, years, mean (SD)	36.30 (12.75)	39.28 (13.09)	37.19 (12.93)	56.27 (8.16)	56.58 (7.93)	56.39 (8.07)
Gender, %						
Female	56.0	67.0	59.3	49.8	61.5	54.4
Male	44.0	33.0	40.7	50.2	38.5	45.6
Marital status, %						
Single	36.7	35.0	36.2	15.4	19.4	17.0
Married or living with partner	52.4	50.0	51.7	57.1	49.6	54.2
Separated, divorced, or widow(er)	11.0	15.0	12.2	27.4	30.9	28.8
Education, %						
No high school diploma or GED	28.1	30.4	28.8	36.5	40.6	38.1
At most a high school diploma or GED	29.4	29.2	29.3	21.0	21.1	21.0
> High school diploma or GED	42.5	40.4	41.9	42.5	38.3	40.8
BMI, kg/m^2^, mean (SD)	28.06 (5.56)	28.85 (6.11)	28.30 (5.74)	29.60 (5.21)	29.95 (5.73)	29.74 (5.42)
Employment, %						
Retired and not currently employed	2.3	4.1	2.8	18.9	20.7	19.6
Not retired and not currently employed	39.2	44.8	40.9	30.4	41.3	34.7
Employed part-time	20.5	18.7	20.0	15.1	13.8	14.6
Employed full-time	38.0	32.4	36.3	35.5	24.2	31.1

Means, percentages and SEs are weighted by OSW. Totals are unweighted. Individuals with missing values on predictors and outcomes have been removed. Additionally, individuals with baseline hypertension have been removed from the incident hypertension sample.

*BMI* body mass index, *HCHS/SOL* Hispanic Community Health Study/Study of Latinos, *MCI* mild cognitive impairment, *OSW* original survey weights.

### Outcomes

Outcomes of interest are incident hypertension, an average of 6 years after the baseline exam, and prevalent MCI, an average of 7 years after the baseline exam. Hypertension (≥ Stage 1) was operationalized as systolic blood pressure ≥ 130 mmHg, DBP ≥ 80 mmHg or use of antihypertensive medications. MCI was according to the National Institute on Aging-Alzheimer’s Association criteria and included individuals with severe impairment/suspect dementia^26^.

### Analyses

For each outcome, we removed any individuals with missing values on the predictors or outcome (at baseline or visit 2). For incident hypertension, we additionally removed individuals with hypertension at baseline. Our final samples sizes for the prevalent MCI and incident hypertension samples are 6,086 and 6,097, respectively. We applied all the weighting and matching-based causal inference approaches to both samples.

### Results

**Figure 5:**
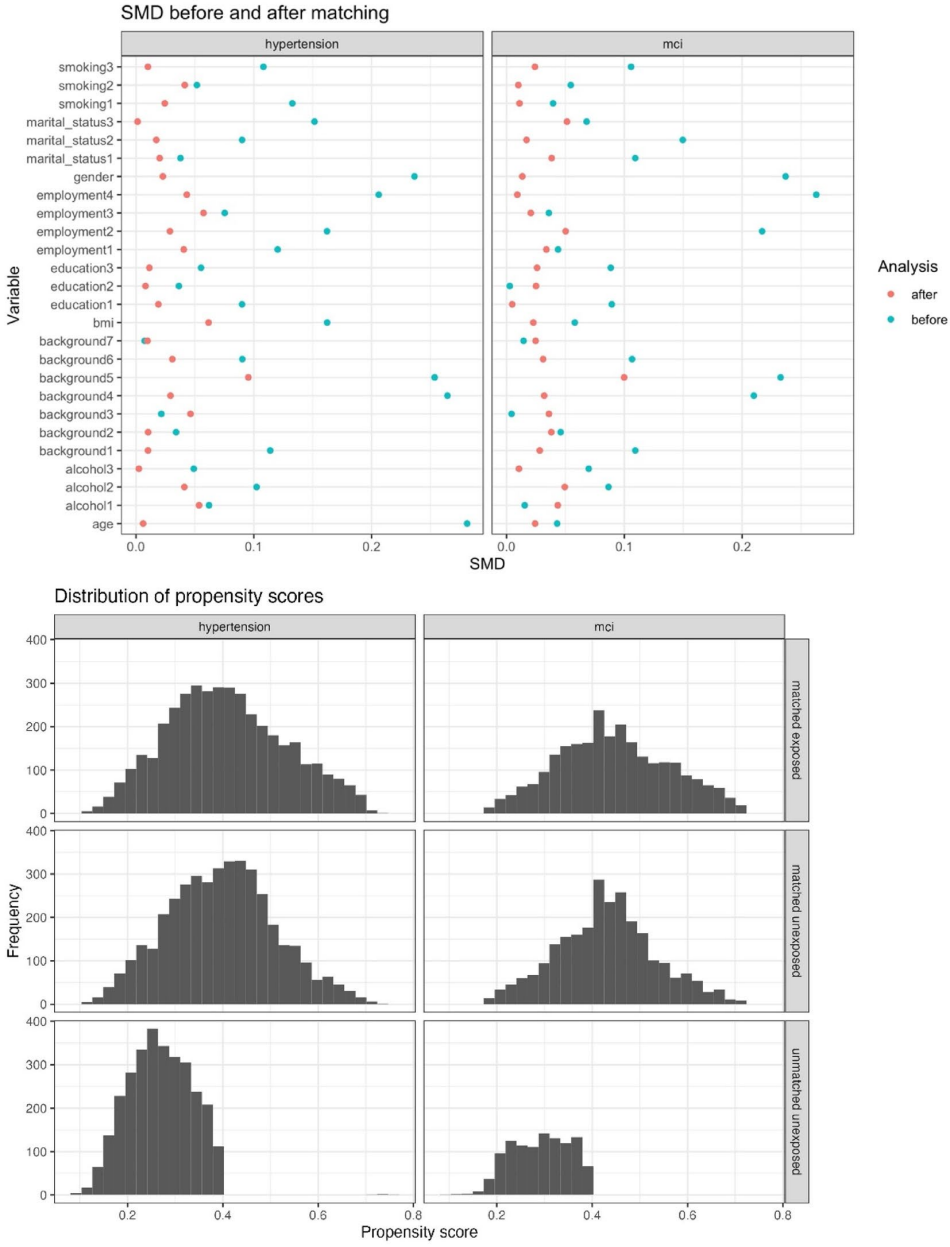
Graphical diagnostics to assess robust PSM method for incident hypertension (left) and prevalent MCI (right) analyses using the HCHS/SOL data. Top: Plot of absolute SMD before and after matching. Bottom: Distribution of propensity scores of matched exposed, matched unexposed and unmatched unexposed individuals. Note that the “unmatched exposed” category is empty because all exposed individuals were matched. *MCI* mild cognitive impairment, *SMD* standardized mean difference.

Supplementary Tables 3 and 4 provide the HCHS/SOL analysis results across all weighting and matching-based causal inference approaches, while Table 6 highlights the results among the robust matching methods only. Comparing individuals with and without insomnia, Table 6 provides the estimated odds ratios for prevalent MCI seven years after, on average, and the estimated incident rate ratios for incident hypertension an average of 6-years after baseline assessment. Based on the robust PSM method, insomnia has a causal effect on both MCI (marginal OR 1.402, CI [1.095, 1.794]; conditional OR 1.432, CI [1.108, 1.850]) and hypertension (marginal IRR 1.184, CI [1.002, 1.400]; conditional IRR 1.174, CI [1.012, 1.360]). Figure 5 provides a plot of the absolute SMD before and after implementing the robust PSM method for each outcome. The robust PSM method does appear to induce better balance in the covariates. Unlike in the simulations, the estimates from the CEM methods diverge substantially and have wide CIs, compared to the estimates from the PSM and weighting methods. This is due to the small number of individuals who were ultimately used in the analysis after conducting CEM.

**Table 6:** HCHS/SOL data analysis results for both prevalent MCI and incident hypertension across the robust matching-based causal inference approaches.

Outcome	Method	Matching	Adjustment	Weights	# Obs	Est	95% CI
Prevalent MCI	PSM	PS via logistic regression with OSW as covariate	Unadjusted	OSW	5008	1.40	(1.10, 1.79)
			Adjusted		5008	1.43	(1.11, 1.85)
	CEM	Binning via coarsened covariates and OSW	Unadjusted	CEMW × OSW	782	0.90	(0.42, 1.90)
			Adjusted		782	0.99	(0.53, 1.84)
Incident hypertension	PSM	PS via logistic regression with OSW as covariate	Unadjusted	OSW	4085	1.18	(1.00, 1.40)
			Adjusted		4085	1.17	(1.01, 1.36)
	CEM	Binning via coarsened covariates and OSW	Unadjusted	CEMW × OSW	474	0.90	(0.53, 1.54)
			Adjusted		474	1.03	(0.64, 1.63)

The estimates are given as ORs for prevalent MCI and IRRs for incident hypertension.

*CEM* coarsened exact matching, *CEMW* coarsened exact matching weights, *CI* confidence interval; *Cover* coverage, *Est* estimate, *IRR* incident rate ratio, *MCI* mild cognitive impairment, *Obs* observations, *OR* odds ratio, *OSW* original survey weights, *PS* propensity score, *PSM* propensity score matching.

## Discussion

Motivated by our interest in applying matching and weighting-based causal inference methods to complex health survey data, we conducted a simulation study to compare various approaches to incorporating the survey weights and design into these methods. We found that most weighting and matching methods performed well under correct specification. However, when a variable (age, in our simulations) was treated as an unmeasured confounder and not included in the matching and effect estimation models (i.e., under-specification) and the survey weights were constructed to depend on this variable, only the matching methods that used the survey weights in both the causal estimation and as a covariate in the matching step continued to perform well. Although age was specifically modelled in simulating the survey weights, our analysis was motivated by the potential for unmeasured variables that are related to demographic or socioeconomic status. The HCHS/SOL survey sampling design accounted for socioeconomic status, yet not all potential sociocultural variables were measured. Thus, it is plausible that an unmeasured variable influenced the sampling process that is nonetheless captured to some extent by the survey weights. As another assessment, we also considered a confounding variable (education in our simulations) that is associated with a design variable (age in simulations). When education was treated as an unmeasured confounder, we saw that the higher its correlation is with the design variable, the better the performance of the robust methods in estimating the causal effects (however confounding bias remains due to imperfect correlation between the unmeasured confounding with the design variable). Therefore, the simulation results suggest that incorporating the survey weights as a covariate in the matching may provide some protection against unmeasured confounding. We recommend further that investigators subsequently incorporate the survey weights in causal effect estimation.

Previous studies have agreed that survey weights should be incorporated in the causal effect estimation step but have disagreed on whether and how to incorporate the survey weights in the matching step. Ridgeway et al. recommended using a survey-weighted propensity score model, while Dugoff et al. concluded that survey weights should be included as a covariate in the propensity score model instead, aligning with our recommendation^11,13^. In contrast, Austin et al. and Lenis et al. found that whether and how the survey weights were incorporated in matching did not impact performance of the method^12,15^. Our study is an important contribution to existing literature. First, while previous studies have focused on continuous outcomes, our study focuses on binary outcomes, targeting both prevalent and incident population estimates of the OR and IRR, respectively. Second, our study is the first to consider the use of CEM in the context of complex survey data. Third, while other studies have used simple sampling designs that are not often employed in practice, our study uses a more complex sampling design and is the first to allow the survey weights to depend on a confounder. Fourth, our study assesses both sensitivity to the introduction of unmeasured confounding and to changes in the exposure and outcome prevalences.

When applying our robust PSM methods that consistently performed well in the simulation study to the HCHS/SOL data, we found that insomnia has a causal association with both prevalent MCI 7 years later and with incident hypertension 6 years later in the US Hispanic/Latino population. Our incident hypertension results support those reported by Li et al.^27^ who estimated the odds ratio for incident hypertension comparing individuals with and without insomnia via logistic regression. In addition, we also provide new evidence of an association between insomnia and prevalent MCI in US Hispanic/Latino adults. We also found that our robust CEM methods performed poorly when applied to the HCHS/SOL data, despite consistently performing well in the simulation study, because of the huge reductions in sample size incurred from matching on a large number of strata. This suggests that CEM may not be practical for small/medium sample sizes and when there are many variables to match on.

Recent sleep research has prioritized using Mendelian Randomization (MR) to conduct causal inference for sleep exposures on downstream health outcomes^28–40^ using genetic variants as instruments for modifiable exposures^1^. However, MR has limitations that have been overshadowed in the wake of its popularity. Violations of MR’s assumptions—relevance, exchangeability, exclusion restriction and homogeneous and linear associations—can result from issues, such as residual pleiotropy, population stratification, linkage disequilibrium, weak IVs and heterogeneity^1,41^. Additionally, lack of relevant genetic variants for the exposure may reduce power for finding causal associations^5^. Specific exposures used by MR studies are also restricted by the specific measures targeted by genome-wide association studies (GWAS) performed. Lastly, most MR studies conducted so far on sleep exposures have used genetic information from predominately European populations, minimizing their generalizability to racial and ethnic minority groups^5^. These limitations of MR underscore the importance of triangulating causal inference from multiple methods currently underutilized in sleep research.

Although we performed an extensive simulation study, there is still room for further investigation in applying causal inference methods to complex health survey data. Future work may focus on––but is not limited to––identifying the best approaches to incorporating the survey weights and design when assessing matching, evaluating robustness of the matching methods after introduction of different types of missingness, assessing the effectiveness of other propensity score estimation approaches and matching algorithms, studying the effect of over-specification of the propensity score and the causal effect estimation models by including unnecessary variables on inference, and investigating other causal inference methods that are not based on weighting or matching.

## Supplementary Information

Supplementary Information.

## Data Availability

HCHS/SOL data are available on the National Heart Lung and Blood Institute’s BioLINCC (Biologic Specimen and Data Repository Information Coordinating Center) repository under accession number HLB01141422a. Alternatively, the data can also be obtained via a data use agreement with the HCHS/SOL Data Coordinating Center at the University of North Carolina at Chapel Hill, see collaborators website: https://sites.cscc.unc.edu/hchs/.

## Abbreviations

ATE: Average treatment effect
ATT: Average treatment effect for the treated
BG: Block group
CATE: Conditional average treatment effect
CATT: Conditional average treatment effect for the treated
CEM: Coarsened exact matching
CEMW: Coarsened exact matching weights
CI: Confidence interval
Cover: Coverage
HCHS/SOL: Hispanic Community Health Study/Study of Latinos
HH: Household
IPTW: Inverse probability of treatment weighting
IRR: Incidence rate ratio
ISW: Inherited survey weights
MCI: Mild cognitive impairment
MR: Mendelian Randomization
Obs: Observations
OR: Odds ratio
OSW: Original survey weights
PS: Propensity score
PSM: Propensity score matching
PSW: Propensity score weighting
RCT: Randomized controlled trial
SE: Standard error
SMD: Standardized mean difference
SUTVA: Stable unit treatment value assumption

## Code availability

Code used for simulations and data analysis is publicly available on the GitHub repository: https://github.com/anjashahu/causal_matching_paper.

## References

[1] Lawlor DA, Harbord RM, Sterne JAC, Timpson N, Davey SG (2008). Mendelian randomization: Using genes as instruments for making causal inferences in epidemiology. Stat. Med..

[2] Rochon PA, Mashari A, Cohen A, Misra A, Laxer D, Streiner DL (2004). The inclusion of minority groups in clinical trials: Problems of under representation and under reporting of data. Account Res..

[3] Faraoni D, Schaefer ST (2016). Randomized controlled trials vs observational studies: Why not just live together?. BMC Anesthesiol..

[4] Pack AI, Magalang UJ, Singh B, Kuna ST, Keenan BT, Maislin G (2021). Randomized clinical trials of cardiovascular disease in obstructive sleep apnea: Understanding and overcoming bias. Sleep.

[5] Sofer T, Goodman MO, Bertisch SM, Redline S (2021). Longer sleep improves cardiovascular outcomes: Time to make sleep a priority. Eur. Heart J..

[6] Munafò MR, Davey SG (2018). Robust research needs many lines of evidence. Nature.

[7] Smart A, Harrison E (2017). The under-representation of minority ethnic groups in UK medical research. Ethn. Health.

[8] McGrath RP, Snih SA, Markides KS, Faul JD, Vincent BM, Hall OT (2019). The burden of health conditions across race and ethnicity for aging Americans: Disability-adjusted life years. Medicine.

[9] Lohr S (2010). Sampling: Design and Analysis.

[10] Stuart EA (2010). Matching methods for causal inference: A review and a look forward. Stat. Sci..

[11] Dugoff EH, Schuler M, Stuart EA (2014). Generalizing observational study results: Applying propensity score methods to complex surveys. Health Serv. Res..

[12] Austin PC, Jembere N, Chiu M (2018). Propensity score matching and complex surveys. Stat. Methods Med. Res..

[13] Ridgeway G, Kovalchik SA, Griffin BA, Kabeto MU (2015). Propensity score analysis with survey weighted data. J. Causal Inference.

[14] Lenis D, Ackerman B, Stuart EA (2018). Measuring model misspecification: Application to propensity score methods with complex survey data. Comput. Stat. Data Anal..

[15] Lenis D, Nguyen TQ, Dong N, Stuart EA (2019). It’s all about balance: Propensity score matching in the context of complex survey data. Biostatistics.

[16] Imbens GW (2004). Nonparametric estimation of average treatment effects under exogeneity: A review. Rev. Econ. Stat..

[17] Hernán MA (2004). A definition of causal effect for epidemiological research. J. Epidemiol. Community Health.

[18] Austin PC, Stuart EA (2015). Moving towards best practice when using inverse probability of treatment weighting (IPTW) using the propensity score to estimate causal treatment effects in observational studies. Stat. Med..

[19] Iacus SM, King G, Porro G (2009). cem: Software for coarsened exact matching. J. Stat. Softw..

[20] King, G. *An Explanation for CEM Weights*. https://docs.google.com/document/d/1xQwyLt_6EXdNpA685LjmhjO20y5pZDZYwe2qeNoI5dE/edit (2012) (Accessed 3 July 2021).

[21] Harder VS, Stuart EA, Anthony JC (2010). Propensity score techniques and the assessment of measured covariate balance to test causal associations in psychological research. Psychol. Methods.

[22] Cai J (2023). Comparisons of Statistical Methods for Handling Attrition in a Follow-up Visit with Complex Survey Sampling. Stat. in Med..

[23] Sorlie PD, Avilés-Santa LM, Wassertheil-Smoller S, Kaplan RC, Daviglus ML, Giachello AL (2010). Design and implementation of the Hispanic Community Health Study/Study of Latinos. Ann. Epidemiol..

[24] Lavange LM, Kalsbeek WD, Sorlie PD, Avilés-Santa LM, Kaplan RC, Barnhart J (2010). Sample design and cohort selection in the Hispanic Community Health Study/Study of Latinos. Ann. Epidemiol..

[25] Levine DW, Kripke DF, Kaplan RM, Lewis MA, Naughton MJ, Bowen DJ (2003). Reliability and validity of the Women’s health initiative insomnia rating scale. Psychol. Assess..

[26] González HM, Tarraf W, Fornage M, González KA, Chai A, Youngblood M (2019). A research framework for cognitive aging and Alzheimer’s disease among diverse US Latinos: Design and implementation of the Hispanic Community Health Study/Study of Latinos-Investigation of Neurocognitive Aging (SOL-INCA). Alzheimers Dement..

[27] Li X, Sotres-Alvarez D, Gallo LC, Ramos AR, Aviles-Santa L, Perreira KM (2021). Associations of sleep-disordered breathing and insomnia with incident hypertension and diabetes. The Hispanic Community Health Study/Study of Latinos. Am. J. Respir. Crit. Care Med..

[28] Ai S, Zhang J, Zhao G, Wang N, Li G, So H-C (2021). Causal associations of short and long sleep durations with 12 cardiovascular diseases: Linear and nonlinear Mendelian randomization analyses in UK Biobank. Eur. Heart J..

[29] Liao L-Z, Li W-D, Liu Y, Li J-P, Zhuang X-D, Liao X-X (2020). Causal assessment of sleep on coronary heart disease. Sleep Med..

[30] van Oort S, Beulens JWJ, van Ballegooijen AJ, Handoko ML, Larsson SC (2020). Modifiable lifestyle factors and heart failure: A Mendelian randomization study. Am. Heart J..

[31] Zhuang Z, Gao M, Yang R, Li N, Liu Z, Cao W (2020). Association of physical activity, sedentary behaviours and sleep duration with cardiovascular diseases and lipid profiles: A Mendelian randomization analysis. Lipids Health Dis..

[32] Daghlas I, Dashti HS, Lane J, Aragam KG, Rutter MK, Saxena R (2019). Sleep duration and myocardial infarction. J. Am. Coll. Cardiol..

[33] Richmond RC, Anderson EL, Dashti HS, Jones SE, Lane JM, Strand LB (2019). Investigating causal relations between sleep traits and risk of breast cancer in women: Mendelian randomisation study. BMJ.

[34] Titova OE, Michaëlsson K, Vithayathil M, Mason AM, Kar S, Burgess S (2021). Sleep duration and risk of overall and 22 site-specific cancers: A Mendelian randomization study. Int. J. Cancer.

[35] Gao X-L, Jia Z-M, Zhao F-F, An D-D, Wang B, Cheng E-J (2020). Obstructive sleep apnea syndrome and causal relationship with female breast cancer: A Mendelian randomization study. Aging (Albany, NY).

[36] Henry A, Katsoulis M, Masi S, Fatemifar G, Denaxas S, Acosta D (2019). The relationship between sleep duration, cognition and dementia: A Mendelian randomization study. Int. J. Epidemiol..

[37] Anderson EL, Richmond RC, Jones SE, Hemani G, Wade KH, Dashti HS (2020). Is disrupted sleep a risk factor for Alzheimer’s disease? Evidence from a two-sample Mendelian randomization analysis. Int. J. Epidemiol..

[38] Gao X, Sun H, Zhang Y, Liu L, Wang J, Wang T (2020). Investigating causal relations between sleep-related traits and risk of type 2 diabetes mellitus: A Mendelian randomization study. Front. Genet..

[39] Dashti HS, Daghlas I, Lane JM, Huang Y, Udler MS, Wang H (2021). Genetic determinants of daytime napping and effects on cardiometabolic health. Nat. Commun..

[40] Daghlas I, Vgontzas A, Guo Y, Chasman DI, Saxena R, International Headache Genetics Consortium (2020). Habitual sleep disturbances and migraine: A Mendelian randomization study. Ann. Clin. Transl. Neurol..

[41] Burgess S, Davey Smith G, Davies NM, Dudbridge F, Gill D, Glymour MM (2020). Guidelines for performing Mendelian randomization investigations. Wellcome Open Res..

