# Supplementary material for: Method comparison and estimation of causal effects of insomnia on health outcomes in a survey sampled population
Source: Sci Rep. 2023 Jun 17;13:9831. doi: 10.1038/s41598-023-36927-2 (PMC10276808; doi:10.1038/s41598-023-36927-2)
Supplement: Supplementary file 1 — Supplementary Information. [file 41598_2023_36927_MOESM1_ESM.pdf]

Method comparison and estimation of causal effects of insomnia on health outcomes in a survey sampled population: the HCHS/SOL

Supplementary Information

Shahu et al.

Assumptions, limitations, and strengths of matching and weighting methods 2
Causal assumptions 2
Limitations 2
Strengths 3
Closed form solutions to estimate the true marginal causal effects in simulation study 3
Tables/Figures 5
Figure 1: Flowchart illustrating sampling design from one sampled dataset for scenario 2, where survey weights are no longer constructed to depend on the confounder, age. 5
Table 1: Simulation results for estimating effect of insomnia on prevalent MCI using weighting methods in the two compared scenarios. 6
Table 2: Simulation results for estimating effect of insomnia on incident hypertension using weighting methods in the two compared scenarios. 7
Table 3: Complete HCHS/SOL data analysis results for prevalent MCI.....................................8
Table 4: Complete HCHS/SOL data analysis results for incident hypertension. 9

Assumptions, limitations, and strengths of matching and weighting methods

We refer the readers to (1,2) for more details, and provide summarized lists below:

Causal assumptions

1. Exchangeability – exposure status is independent of potential outcomes given covariates
2. Positivity – no combination of covariates is unique to an exposure group because there is sufficient overlap of covariates across exposure groups and individuals have the potential to receive any exposure
3. Stable Unit Treatment Value Assumption (SUTVA) – an individual's outcome depends only on that individual's exposure status and not on the exposure status of others

Limitations

1. Existence of unmeasured covariates that are unrelated to measured covariates, spillover effects, etc. may violate causal assumptions, leading to biased results
2. Valid inference using PSM and weighting may rely on correct specification of propensity score model
3. Valid inference using doubly robust methods relies on correct specification of the outcome model
4. PSM and CEM may discard individuals, leading to loss of information and reduced power

Strengths

1. Addresses limitations of conventional observational analyses by minimizing confounding

2. Addresses limitations of RCTs by being less resource-intensive and potentially more generalizable

3. Straightforward metrics are available to assess performance of matching method in terms of balance and overlap of exposed and unexposed groups

4. Can be used in combination with regression techniques (i.e. doubly robust methods)

Closed form solutions to estimate the true marginal causal effects in simulation study

Unlike the conditional causal effects, closed form solutions are available for the marginal causal effects. For prevalent MCI (on the odds ratio scale), we let $\Pr[Y(0)=1]=\frac{1}{N}\sum_{i=1}^{N}Y_i(0)$ and $\Pr[Y(1)=1]=\frac{1}{N}\sum_{i=1}^{N}Y_i(1)$ for the ATE. We let $\Pr[Y_{Z=1}(0)=1]=\frac{1}{N_{Z=1}}\sum_{i=1}^{N_{Z=1}}Y_i(0)$ and $\Pr[Y_{Z=1}(1)=1]=\frac{1}{N_{Z=1}}\sum_{i=1}^{N_{Z=1}}Y_i(1)$ for the ATT. The closed form solutions are as follows:

$$\text{ATE:}\ \frac{\frac{Pr[Y(1)=1]}{1-Pr[Y(1)=1]}}{\frac{Pr[Y(0)=1]}{1-\ Pr[Y(0)=1]}}$$

$$\text{ATT: } \frac{\frac{Pr[Y_{Z=1}(1) = 1]}{1 - Pr[Y_{Z=1}(1) = 1]}}{\frac{Pr[Y_{Z=1}(0) = 1]}{1 - Pr[Y_{Z=1}(0) = 1]}}$$

For incident hypertension (on the incident rate ratio scale), we let t refer to the years between visits. The closed form solutions are as follows:

$$\text{ATE: } \frac{\frac{\sum_{i=1}^{N_{Y_1(1)=0}} Y_{i2}(1)}{\sum_{i=1}^{N_{Y_1(1)=0}} \mathrm{t}}}{\frac{\sum_{i=1}^{N_{Y_1(0)=0}} Y_{i2}(0)}{\sum_{i=1}^{N_{Y_1(0)=0}} \mathrm{t}}}$$

$$\text{ATT: } \frac{\frac{\sum_{i=1}^{N_{Y_1(1)=0,\mathrm{Z}=1}} Y_{i2}(1)}{\sum_{i=1}^{N_{Y_1(1)=0,\mathrm{Z}=1}} \mathrm{t}}}{\frac{\sum_{i=1}^{N_{Y_1(0)=0,\mathrm{Z}=1}} Y_{i2}(0)}{\sum_{i=1}^{N_{Y_1(0)=0,\mathrm{Z}=1}} \mathrm{t}}}$$

Tables/Figures

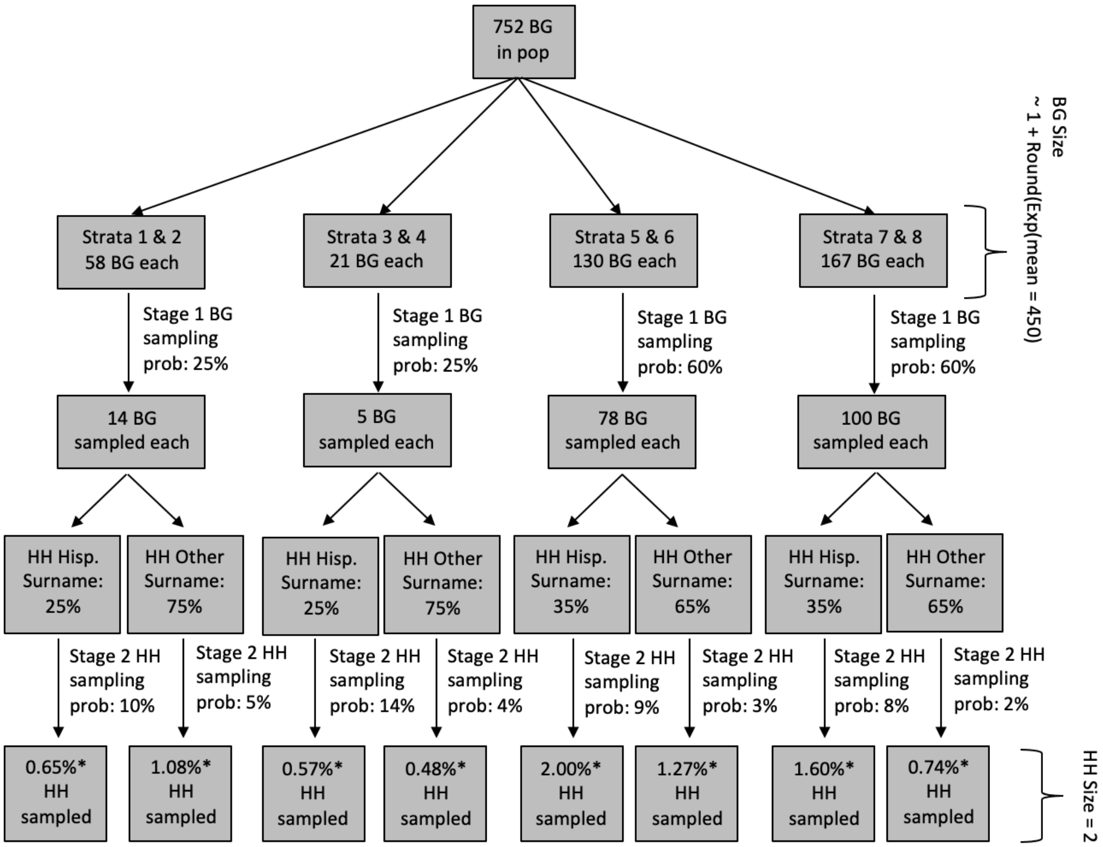

* % of HH sampled will vary due to randomness in sampling

Figure 1: Flowchart illustrating sampling design from one sampled dataset for scenario 2, where survey weights are no longer constructed to depend on the confounder, age.

				Scenario 1		Scenario 2	
Specification	PS	Adjustment	PSW	Bias	Cover	Bias	Cover
Correct	Via weighted logistic regression using OSW	Unadjusted	IPTW	-0.024	0.931	0.010	0.951
			IPTW x OSW	0.010	0.946	0.012	0.953
			ODDS	-0.016	0.950	0.004	0.952
			ODDS x OSW	0.007	0.950	0.009	0.945
		Adjusted	IPTW	-0.026	0.946	0.010	0.949
			IPTW x OSW	0.010	0.938	0.014	0.949
			ODDS	-0.020	0.960	0.005	0.954
			ODDS x OSW	0.010	0.935	0.011	0.948
	Via logistic regression with OSW as covariate	Unadjusted	IPTW	-0.021	0.958	0.009	0.956
			IPTW x OSW	0.009	0.947	0.011	0.948
			ODDS	-0.013	0.969	0.003	0.955
			ODDS x OSW	0.009	0.947	0.008	0.946
		Adjusted	IPTW	-0.026	0.946	0.010	0.949
			IPTW x OSW	0.010	0.938	0.014	0.950
			ODDS	-0.020	0.958	0.005	0.955
			ODDS x OSW	0.010	0.936	0.011	0.948
Under (no age)	Via weighted logistic regression using OSW	Unadjusted	IPTW	0.128	0.484	0.267	0.142
			IPTW x OSW	0.270	0.513	0.271	0.273
			ODDS	0.137	0.426	0.267	0.147
			ODDS x OSW	0.275	0.511	0.273	0.258
		Adjusted	IPTW	0.106	0.614	0.245	0.205
			IPTW x OSW	0.248	0.588	0.250	0.346
			ODDS	0.115	0.556	0.245	0.211
			ODDS x OSW	0.252	0.571	0.251	0.347
	Via logistic regression with OSW as covariate	Unadjusted	IPTW	0.053	0.890	0.267	0.140
			IPTW x OSW	-0.145	0.848	0.271	0.273
			ODDS	0.071	0.804	0.268	0.149
			ODDS x OSW	0.007	0.944	0.273	0.264
		Adjusted	IPTW	0.031	0.939	0.245	0.206
			IPTW x OSW	-0.163	0.810	0.249	0.350
			ODDS	0.048	0.892	0.245	0.210
			ODDS x OSW	-0.016	0.938	0.251	0.351

Table 1: Simulation results for estimating effect of insomnia on prevalent MCI using weighting methods in the two compared scenarios. Scenario 1: survey weights depend on age. Scenario 2: survey weights do not depend on age. Bias: difference between the causal effect estimate obtained from the full (unsampled) simulated target population dataset and the average estimated causal effect over the 1,000 survey samples. Cover: coverage of the 95% confidence intervals (CIs) defined as the proportion of simulations in which the true effect is covered by the CIs.

				Scenario 1		Scenario 2	
Specification	PS	Adjustment	PSW	Bias	Cover	Bias	Cover
Correct	Via weighted logistic regression using OSW	Unadjusted	IPTW	-0.041	0.710	-0.006	0.951
			IPTW x OSW	-0.002	0.950	-0.003	0.958
			ODDS	-0.038	0.708	-0.004	0.961
			ODDS x OSW	-0.004	0.955	-0.002	0.960
		Adjusted	IPTW	-0.036	0.737	-0.004	0.955
			IPTW x OSW	-0.001	0.936	-0.002	0.958
			ODDS	-0.030	0.771	-0.002	0.960
			ODDS x OSW	0.001	0.936	0.001	0.962
	Via logistic regression with OSW as covariate	Unadjusted	IPTW	-0.040	0.729	-0.006	0.954
			IPTW x OSW	-0.003	0.946	-0.004	0.954
			ODDS	-0.036	0.740	-0.005	0.959
			ODDS x OSW	-0.003	0.947	-0.002	0.954
		Adjusted	IPTW	-0.036	0.735	-0.004	0.956
			IPTW x OSW	-0.001	0.934	-0.002	0.958
			ODDS	-0.030	0.777	-0.002	0.960
			ODDS x OSW	0.001	0.936	0.001	0.962
Under (no age)	Via weighted logistic regression using OSW	Unadjusted	IPTW	0.041	0.723	0.118	0.163
			IPTW x OSW	0.122	0.675	0.121	0.262
			ODDS	0.045	0.677	0.121	0.120
			ODDS x OSW	0.123	0.675	0.124	0.223
		Adjusted	IPTW	0.043	0.692	0.123	0.131
			IPTW x OSW	0.125	0.662	0.125	0.232
			ODDS	0.049	0.608	0.126	0.095
			ODDS x OSW	0.127	0.653	0.128	0.192
	Via logistic regression with OSW as covariate	Unadjusted	IPTW	-0.021	0.913	0.119	0.160
			IPTW x OSW	-0.134	0.816	0.121	0.259
			ODDS	-0.005	0.946	0.121	0.123
			ODDS x OSW	-0.041	0.902	0.124	0.213
		Adjusted	IPTW	-0.022	0.900	0.123	0.129
			IPTW x OSW	-0.140	0.794	0.125	0.232
			ODDS	-0.004	0.950	0.126	0.093
			ODDS x OSW	-0.045	0.892	0.129	0.190

Table 2: Simulation results for estimating effect of insomnia on incident hypertension using weighting methods in the two compared scenarios. Scenario 1: survey weights depend on age. Scenario 2: survey weights do not depend on age. Bias: difference between the causal effect estimate obtained from the full (unsampled) simulated target population dataset and the average estimated causal effect over the 1,000 survey samples. Cover: coverage of the 95% confidence intervals (CIs) defined as the proportion of simulations in which the true effect is covered by the CIs.

Method	Matching / PS	Adjustment	Weights	# Obs	OR	95% CI
PSM	PS via weighted logistic regression using OSW	Unadjusted	No weights	5009	1.266	(1.063, 1.508)
			OSW	5009	1.411	(1.095, 1.818)
			ISW	5009	1.496	(1.166, 1.920)
		Adjusted	No weights	5009	1.230	(1.030, 1.471)
			OSW	5009	1.412	(1.089, 1.831)
			ISW	5009	1.359	(1.060, 1.743)
	PS via logistic regression with OSW as covariate	Unadjusted	No weights	5008	1.293	(1.085, 1.542)
			OSW	**5008**	**1.402**	**(1.095, 1.794)**
			ISW	5008	1.501	(1.184, 1.902)
		Adjusted	No weights	5008	1.285	(1.076, 1.538)
			OSW	**5008**	**1.432**	**(1.108, 1.850)**
			ISW	5008	1.386	(1.098, 1.749)
CEM	Binning via coarsened covariates	Unadjusted	CEMW	1540	1.429	(1.030, 1.983)
			CEMW x OSW	1540	1.538	(0.988, 2.394)
		Adjusted	CEMW	1540	1.444	(1.027, 2.030)
			CEMW x OSW	1540	1.691	(1.097, 2.604)
	Binning via coarsened covariates & OSW	Unadjusted	CEMW	782	1.129	(0.718, 1.775)
			CEMW x OSW	**782**	**0.896**	**(0.423, 1.901)**
		Adjusted	CEMW	782	1.113	(0.706, 1.755)
			CEMW x OSW	**782**	**0.992**	**(0.534, 1.843)**
Weighting	PS via weighted logistic regression using OSW	Unadjusted	IPTW	6086	1.292	(1.088, 1.535)
			IPTW x OSW	6086	1.420	(1.113, 1.812)
			ODDS	6086	1.276	(1.071, 1.520)
			ODDS x OSW	6086	1.384	(1.091, 1.755)
		Adjusted	IPTW	6086	1.274	(1.071, 1.517)
			IPTW x OSW	6086	1.452	(1.134, 1.861)
			ODDS	6086	1.265	(1.060, 1.510)
			ODDS x OSW	6086	1.427	(1.118, 1.822)
	PS via logistic regression with OSW as covariate	Unadjusted	IPTW	6086	1.265	(1.064, 1.504)
			IPTW x OSW	6086	1.395	(1.083, 1.797)
			ODDS	6086	1.261	(1.058, 1.503)
			ODDS x OSW	6086	1.351	(1.069, 1.708)
		Adjusted	IPTW	6086	1.278	(1.073, 1.523)
			IPTW x OSW	6086	1.450	(1.126, 1.867)
			ODDS	6086	1.281	(1.072, 1.530)
			ODDS x OSW	6086	1.416	(1.117, 1.794)

Table 3: Complete HCHS/SOL data analysis results for prevalent MCI. Results are provided across all the considered weighting and matching-based causal inference approaches. The four matching methods that we consider robust based on results from simulations with under specification are highlighted with bold text.

Method	Matching / PS	Adjustment	Weights	# Obs	IRR	95% CI
PSM	PS via weighted logistic regression using OSW	Unadjusted	No weights	4089	1.089	(0.969, 1.224)
			OSW	4089	1.131	(0.954, 1.340)
			ISW	4089	1.045	(0.890, 1.227)
		Adjusted	No weights	4089	1.077	(0.958, 1.211)
			OSW	4089	1.137	(0.982, 1.316)
			ISW	4089	1.140	(0.985, 1.320)
	PS via logistic regression with OSW as covariate	Unadjusted	No weights	4085	1.089	(0.969, 1.224)
			OSW	**4085**	**1.184**	**(1.002, 1.400)**
			ISW	4085	1.055	(0.897, 1.240)
		Adjusted	No weights	4085	1.076	(0.957, 1.209)
			OSW	**4085**	**1.174**	**(1.012, 1.360)**
			ISW	4085	1.090	(0.945, 1.257)
CEM	Binning via coarsened covariates	Unadjusted	CEMW	1053	1.007	(0.792, 1.279)
			CEMW x OSW	1053	1.092	(0.724, 1.647)
		Adjusted	CEMW	1053	0.966	(0.769, 1.212)
			CEMW x OSW	1053	1.092	(0.778, 1.531)
	Binning via coarsened covariates & OSW	Unadjusted	CEMW	474	0.851	(0.598, 1.210)
			CEMW x OSW	**474**	**0.902**	**(0.529, 1.540)**
		Adjusted	CEMW	474	0.870	(0.617, 1.228)
			CEMW x OSW	**474**	**1.025**	**(0.645, 1.630)**
Weighting	PS via weighted logistic regression using OSW	Unadjusted	IPTW	6097	1.049	(0.951, 1.157)
			IPTW x OSW	6097	1.077	(0.915, 1.268)
			ODDS	6097	1.048	(0.954, 1.152)
			ODDS x OSW	6097	1.089	(0.932, 1.274)
		Adjusted	IPTW	6097	1.043	(0.953, 1.141)
			IPTW x OSW	6097	1.085	(0.937, 1.257)
			ODDS	6097	1.055	(0.967, 1.151)
			ODDS x OSW	6097	1.107	(0.966, 1.269)
	PS via logistic regression with OSW as covariate	Unadjusted	IPTW	6097	1.058	(0.960, 1.166)
			IPTW x OSW	6097	1.080	(0.912, 1.280)
			ODDS	6097	1.059	(0.964, 1.165)
			ODDS x OSW	6097	1.100	(0.950, 1.274)
		Adjusted	IPTW	6097	1.041	(0.952, 1.139)
			IPTW x OSW	6097	1.090	(0.934, 1.273)
			ODDS	6097	1.053	(0.965, 1.148)
			ODDS x OSW	6097	1.108	(0.972, 1.263)

Table 4: Complete HCHS/SOL data analysis results for incident hypertension. Results are provided across all the considered weighting and matching-based causal inference approaches. The four matching methods that we consider robust based on results from simulations with under specification are highlighted with bold text.

Bibliography

1. Stuart EA. Matching methods for causal inference: A review and a look forward. Stat Sci. 2010 Feb 1;25(1):1–21.
2. Dugoff EH, Schuler M, Stuart EA. Generalizing observational study results: applying propensity score methods to complex surveys. Health Serv Res. 2014 Feb;49(1):284–303.
